# Weyl Mott Insulator

**DOI:** 10.1038/srep19853

**Published:** 2016-01-29

**Authors:** Takahiro Morimoto, Naoto Nagaosa

**Affiliations:** 1 RIKEN Center for Emergent Matter Science (CEMS), Wako, Saitama 351-0198, Japan; 2 Department of Applied Physics, University of Tokyo, 7-3-1, Hongo, Bunkyo-ku, Tokyo 113-8656, Japan

## Abstract

Relativistic Weyl fermion (WF) often appears in the band structure of three dimensional magnetic materials and acts as a source or sink of the Berry curvature, i.e., the (anti-)monopole. It has been believed that the WFs are stable due to their topological indices except when two Weyl fermions of opposite chiralities annihilate pairwise. Here, we theoretically show for a model including the electron-electron interaction that the Mott gap opens for each WF without violating the topological stability, leading to a topological Mott insulator dubbed *Weyl Mott insulator* (WMI). This WMI is characterized by several novel features such as (i) energy gaps in the angle-resolved photo-emission spectroscopy (ARPES) and the optical conductivity, (ii) the nonvanishing Hall conductance, and (iii) the Fermi arc on the surface with the penetration depth diverging as approaching to the momentum at which the Weyl point is projected. Experimental detection of the WMI by distinguishing from conventional Mott insulators is discussed with possible relevance to pyrochlore iridates.

Weyl fermions (WFs) in solids attract recent intensive interests from the viewpoint of their novel quantum transport properties and chiral anomaly. The WF is described by the 2-component spinors originating from 4-component Dirac spinor when the mass *m* is zero in the Dirac equation. The realization of WFs in condensed matters has been recently established^1^,^2^,^3^. In magnetic materials, the time-reversal symmetry is broken and the energy dispersion of Bloch wavefunction has no Kramer’s degeneracy. In this case, the band crossings between the two bands are described by a 2 × 2 Hamiltonian as


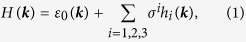


with 2 × 2 Pauli matrices *σ*^*i*^ (*i* = 1, 2, 3). Three conditions of the band crossing *h*_*i*_(***k***) = 0 for (*i* = 1, 2, 3) can be satisfied in general by appropriately choosing the three components of the crystal momentum ***k***. Weyl points sometimes exist exactly at the Fermi energy when dictated by some symmetry and topology of the Bloch wavefunctions, for example, in Dirac semimetals^4^,^5^,^6^,^7^,^8^. More recently, experimental discovery of Weyl semimetals in an inversion broken material TaAs has been reported^9^,^10^,^11^.

Weyl fermion plays an important role in the context of the Berry phase, which is defined by 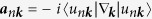 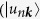 : the periodic part of the Bloch wave function with the band index *n* = ± and the momentum ***k***) and acts as the vector potential in the momentum space. The Berry curvature ***b***_*n**k***_ = ∇_***k***_ × ***a***_*n**k***_ is the emergent magnetic field, and can be enhanced near the band crossing points. When one expand Eq. (1) around the band crossing point (Weyl point, which we assume to be ***k***_0_ = **0**), there appears the WF described with *h*_*i*_(***k***) = *ηv*_*F*_*k*_*i*_, by an appropriate choice of the coordinate *k*_*i*_’s, where *v*_*F*_ is the Fermi velocity. The sign *η* = ±1 specifies the chirality of the WF, and the Berry curvature of the lower eigenstates (*n* = −) of the Hamiltonian in Eq. (1) is obtained as


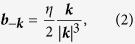


which diverges as |***k***| → 0 and the total flux Φ penetrating the surface *S* enclosing the Weyl point is given by 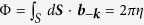. This indicates that the WF acts as the magnetic monopole (anti-monopole) for *η* = 1 (*η* = −1); the magnetic charge 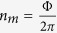 plays a role of topological index. Strong Berry curvature leads to the enhanced anomalous Hall effect^1^ as well as the chiral magnetic effect which results in the negative magneto-resistance^12^.

Figure 1 shows the schematic figure of the three dimensional first Brillouin zone in which two WFs exist along the *k*_*z*_ direction. One can define the Chern number


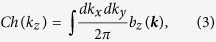


for the plane of fixed *k*_*z*_. When we consider *Ch*(*k*_*z*_) as a function of *k*_*z*_, there appears the jump by *η* at *k*_*z*_ = ±*k*_0*z*_, i.e., *k*_*z*_-coordinate of the Weyl points. Therefore, due to the periodicity of *Ch*(*k*_*z*_) by *k*_*z*_ → *k*_*z*_ + 2*π*/*c*, we need the pair of *η* = 1 and −1^13^,^14^. The existence of a single (an odd number of) WF is also excluded. Therefore, the annihilation of a single WF is prohibited, i.e., the only way to destroy the WFs is to annihilate a pair of WFs with opposite chiralities either by making the two WFs approach to each other in the momentum space or by introducing a scattering between two WFs with some density-wave-type order. The former scenario is actually proposed for the transition between the Weyl metal and insulator in pyrochlore compounds^15^. The latter one is also discussed intensively^16^,^17^. Meanwhile, effects of the electron correlation have been discussed for WFs by several methods including random phase approximation^18^ and, more recently, cluster perturbation theory^19^. However, the possibility of the Mott gap opening at each WF has never been explored thus far to the best of the present authors’ knowledge.

In this paper, we study the effect of the electron correlation *U* on WFs by using a simple model which is exactly solvable. It is shown that the Mott gap due to *U* open at each WF without the pair annihilation, while the topological properties are kept unchanged. Namely, the magnetic charge of the WF is unchanged with the role of poles in Green’s function being replaced by its zeros. The Hall conductance *σ*_*xy*_ remains nonvanishing and the Fermi arc on the surface remains, while the Green’s function and the optical conductivity *σ*_*xx*_(*ω*) show the gap. Therefore, this Mott insulating state is identified as a topological Mott insulator, and we name it “Weyl Mott Insulator (WMI)”. The experimental detection of this new state is also discussed.

## Results

### Model and Green’s function

The model we study is given by the Hamiltonian


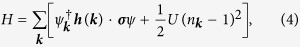


where *ψ*_***k***_ = (*c*_***k***,↑_, *c*_***k***,↓_)^*T*^ is the two-component spinor, and 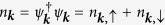. The most peculiar nature of this model arises from the electron-electron interaction which is local in ***k***, i.e., the Hamiltonian is decomposed into independent ***k***-sectors. In the real space, this corresponds to the non-local interaction in the limit of forward scattering. A similar idea has been explored to study the Mott transition^20^ and the spin-charge separation^21^. This locality of the interaction in ***k*** enables the exact solution of this problem. One can introduce the unitary transformation *U*(***k***) satisfying *U*(***k***)^†^[***h***(***k***) ⋅ ***σ***]*U*(***k***) = *σ*^3^*h*(***k***) with *h*(***k***) = |***h***(***k***)| and a new spinor *ϕ*_***k***_ = *U*(***k***)^†^*ψ*_***k***_ = (*a*_***k***+_, *a*_***k***−_)^*T*^, and then obtain


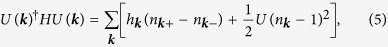


with 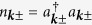 and *n*_***k***_ = *n*_***k***+_ + *n*_***k***−_. There are four eigenstates and eigenenergies: (i) 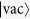 with 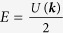, (ii) 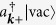 with *E* = *h*(***k***), (iii) 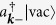 with *E* = −*h*(***k***), and (iv) 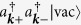 with 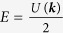.

**Figure 2:**
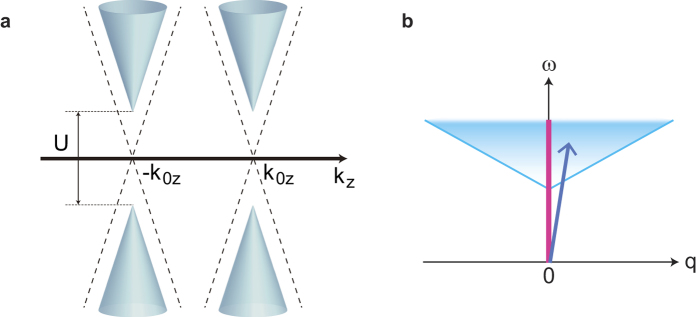
Energy spectrum of WMI. (**a**) Poles of the Green’s function. Dashed line represents the band structure of a noninteracting Weyl semimetal. Energy bands are shifted by ± *U*/2 and show a Mott gap of *U*. (**b**) Excitation spectrum indicating the region of non-zero conductivity *σ*(***q***, *ω*). Excitation gap of *U* + *v*_*F*_|***q***| is finite for nonzero momentum transfer ***q***. The spectrum is singular at ***q***= **0** where gapless excitations are allowed due to *k*-local excitations without the energy cost of *U*.

Using these solutions, one can easily obtain the thermal Green’s function in the zero temperature limit as


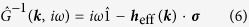


where ***h***_eff_(***k***) = ***n***(***k***)[*h*(***k***) + *U*/2] with ***n***(***k***) = ***h***(***k***)/*h*(***k***). (For details, see Supplementary Information SI.) As can be seen from Eq. (6), the energy dispersions of the poles are *ε*_±_(***k***) = ±*h*(***k***) + *U*/2, where the Mott gap of *U* exists even at the Weyl point with *h*(***k***) = 0 as shown in Fig. 2(a), which can be measured in the angle-resolved photoemission spectroscopy (ARPES). It should be noticed that Eq. (6) is derived from the exact Green’s function and is not a result of some mean-field approximation. Thus, the WFs disappear due to the electron correlation without the pair annihilation.

### Topological properties

The topological index for the interacting electronic systems can be defined in terms of Green’s function^22^. For the (2 + 1)D case, it is given by


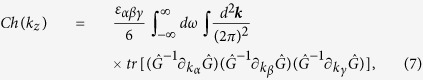


where *α*, *β*, *γ* run over 0, 1, 2, and *ε*_*αβγ*_ is the totally antisymmetric tensor. Plugging Eq. (6) into Eq. (7), one obtains


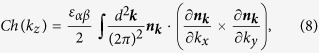


where the *k*-integral is over *k*_*x*_ and *k*_*y*_ for fixed *k*_*z*_. Since ***n***(***k***) = ***h***_eff_(***k***)/|***h***_eff_(***k***)| = ***h***(***k***)/|***h***(***k***)|, *Ch*(*k*_*z*_) does not change in spite of the gap opening at Weyl points. The Green’s function in Eq. (6) depends on the direction in which ***k*** approaches to the Weyl point ***k***_0_ and still plays a role of a source (sink) of the Berry curvature. At exactly ***k***_0_, 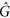 has a zero at *ω* = 0 when one averages over the direction of 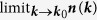. Namely, the role of a pole is replaced by a zero in the topological properties of Green’s function^22^. Because of the bulk-edge correspondence, nonzero topological index *Ch*(*k*_*z*_) indicates that the existence of the surface states on the side surface, i.e., the Fermi arc. (For details, see Supplementary Information SII.)

Therefore, the present insulating state is topological and we call it “Weyl Mott insulator (WMI)” distinct from the usual antiferromagnetic Mott insulator (AFI).

### Optical conductivity

Now we study the conductivity, which is given by the two-particle correlation function. We consider a single WF with the Fermi velocity *v*_*F*_ described by ***h***(***k***) = *v*_*F*_***k***. It is crucial to distinguish between the nonzero momentum transfer ***q*** and exactly ***q*** = **0**. In the former case, the double occupancy of the electrons will be created, while not in the latter case, which brings about the singularity or discontinuity at ***q*** = **0**. This reflects the long-range nature of our Coulomb interaction in Eq. (4). For ***q*** ≠ **0**, the particle-hole continuum starts from *ω* = *U* + *v*_*F*_|***q***| for the transition of an electron from ***k*** to ***k*** + ***q***.

**Figure 3:**
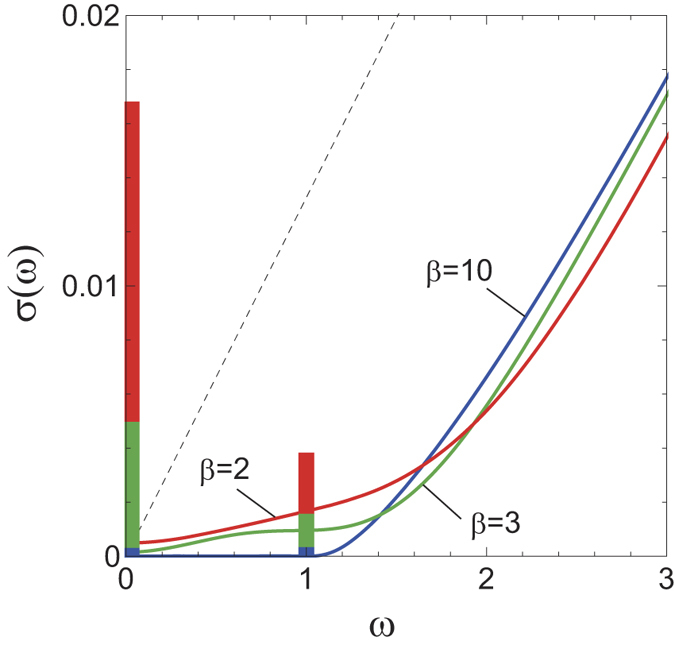
Temperature dependence of the optical conductivity of WMIs. We plotted results for *β* = 10 (blue), *β* = 3 (green), and *β* = 2 (red). The delta functions at *ω* = 0 and *U* from the intraband contributions are denoted by bars whose heights represent mutual ratios of the weights. The dashed line represents *σ*(*ω*) for a free WF. We set *U* = 1.

For the optical conductivity, the momentum of the incident light ***q*** is finite although small, and hence we take the limit ***q*** → **0**. In this limit, the optical conductivity at zero temperature for a single WF is obtained as (See Supplementary Information SIII for details)


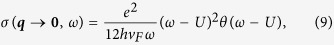


where *θ*(*x*) = 1(*x* ≥ 0) and *θ*(*x*) = 0(*x* < 0). The optical conductivity shows a Mott gap of *U* and its asymptotic behavior for large *ω* is given by 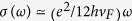 which coincides with the well known result for a free WF^23^. In Fig. 3, the optical conductivity is plotted for various temperatures (See Supplementary Information SIII for the derivation). As the temperature increases, peaks at *ω* = 0 and *ω* = *U* appear, as denoted by bars whose heights represent mutual ratios of the weights of the peaks. The peak at *ω* = 0 is a Drude peak for finite temperatures, while the peak at *ω* = *U* arises from an intraband contribution in which a WF at ***k*** scattered to ***k*** + ***q*** within the same band feels a Coulomb repulsion *U*. The appearance of the peak at *ω* = *U* in *σ*(*ω*) for WMIs contrasts to the absence of such a peak for AFIs, because the peak at *ω* = *U* originates from the correlation effect. In addition, the appearance of the in-gap absorption indicates the fragile nature of the Mott gap compared with the single-particle band gap.

For the case of exactly ***q*** = **0**, only the vertical transitions within the same ***k***-sector contribute to the conductivity as indicated by the red line in Fig. 2(b). Since no double occupancy is created in this case, no energy cost of *U* occurs. Therefore, the conductivity *σ*(***q*** = **0**, *ω*) is given by Eq. (9) with *U* = 0.

## Discussions

**Figure 4:**
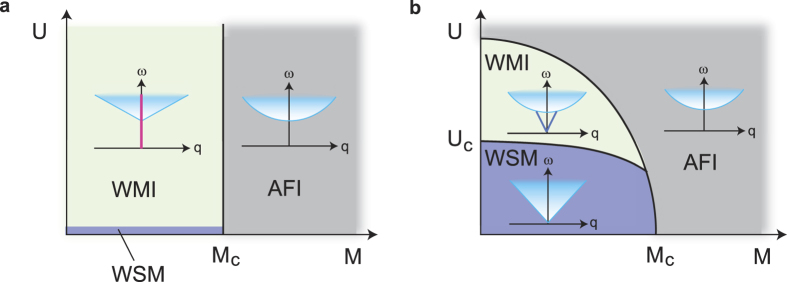
Phase diagrams of the Weyl semimetal. (**a**) The phase diagram of the Hamiltonian in Eq. (4) for WMIs. (**b**) The conjectured phase diagram for Weyl semimetals with more realistic interactions. Insets show the excitation spectra of the conductivity *σ*(***q***, *ω*). The singularity at ***q*** = **0** in the excitation spectrum for the WMI phase turns into a gapless collective excitation for realistic interactions.

Now the relevance of the present results to realistic systems is discussed. There are clear differences between the WMI and the AFI due to their topological nature: (i) The Hall conductance is finite in WMI while it is zero in AFI. (ii) Correspondingly, the Fermi arc on the surface remains in WMI while not in AFI. Also importantly, the phase transition between the WMI and AFI is possible once the former exists as explained below. Figure 4(a) shows the phase diagram of the present model. The horizontal axis is the separation Δ*k*_*z*_ between the two WFs which is controlled by, e.g., the strength of the antiferromagnetic long range order parameter *M*. When *U* = 0, the phase transition occurs from the Weyl semimetal to the AFI by the pair annihilation of WFs at *M* = *M*_*c*_. Once the interaction *U* is switched on, we always opens the gap and the system becomes the WMI as long as Δ*k*_*z*_ is finite. Along the phase transition line Δ*k*_*z*_ = 0 at *M* = *M*_*c*_, *U* > 0, the pair annihilation of the two zeros of the Green’s function occurs, which is distinct from that at *U* = 0 where the two poles collide and pair-annihilate. Here one must consider the peculiarity of the present model. The effect of the long range Coulomb interactions is marginally irrelevant as in the case of quantum electrodynamics (QED)^24^. As for the short-range Coulomb interaction *U*, it is irrelevant. This means that there must be a finite range of Coulomb interaction within which the WFs remains gapless and stable. On the other hand, the strong *U* limit in the lattice model corresponds to the localized electron and hence a trivial AFI. Therefore, the conjectured phase diagram of a more realistic model is given in Fig. 4(b), where the successive transitions from the Weyl semimetal to the WMI, and from the WMI to the AFI occurs as the strength of the interaction increases. (The separation of two WFs is reduced also as the interaction increases and hence the trajectory should goes as *U* and *M* simultaneously increase.)

Now we discuss the Green’s function and the two-particle correlation function for realistic electron-electron interactions given by


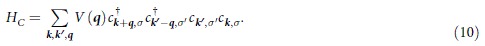


The self-energy Σ(***k*** = **0**, *ω*) of the Green’s function in the second order in *V*(***q***) is given in Supplementary Information SIV. It is concluded that the gap of the spectral function is stable and remains nonzero. The two-particle correlation functions such as *σ*(***q***, *ω*), on the other hand, is gapless at ***q*** = **0** for the Hamiltonian in Eq. (4). For a finite size system of *N* sites, the number of poles forming this gapless excitation in the two particle correlation function [the red line in Fig. 2(b)] is of *O*(*N*) (which is the number of ***k*** where the excitation can be created). In general, the number of poles for a collective excitation is of *O*(*N*), while that of a continuous excitation arising as a pair of single-particle excitations is of *O*(*N*^2^). This infers that the gapless excitations at ***q*** = **0** corresponds to a collective excitation. For realistic interactions, the discontinuity at ***q*** = **0** should be removed. In this case, we conjecture that the vertical transition [red line in the inset of Fig. 4(a)] turns into a collective mode with a linear dispersion as shown in the blue line in the inset in the WMI phase of Fig. 4(b).

These considerations offer a different scenario to interpret the phase diagram of pyrochlore iridates *R*_2_Ir_2_O_7_^3^,^15^. As the radius of the rare-earth ion *R* is reduced, the correlation strength increases. A recent optical measurement in Nd_2_Ir_2_O_7_ has revealed the opening of the Mott gap of the order of 0.05 eV^25^. A transport experiment also discovered the metallic domain wall states even in the Mott insulating state, i.e., the bulk is insulating while the domain wall is metallic^26^. As the correlation is further reduced, these surface metallic states also disappear. One scenario is proposed by Yamaji *et al.*^27^ based on the mean field theory. As an alternative scenario, one can consider the two types of Mott insulators, i.e., the WMI and the AFI, and the disappearance of the metallic domain wall states signals the phase transition between the two phases. Namely, since two domains of the antiferromagnetic order correspond to opposite signs of *σ*_*xy*_ and hence the two-dimensional chiral surface modes are expected to appear at the domain boundary in the WMI phase. However, we note that this requires a symmetry lowering to violate the cancellation of the Chern vectors pointing toward four momentum directions equivalent to (1, 1, 1) which makes *σ*_*xy*_ zero in the cubic symmetric case^28^. The smoking-gun experiment for the WMI should be the ARPES to detect the Fermi arc even in the Mott insulating phase as mentioned above.

## Additional Information

**How to cite this article**: Morimoto, T. and Nagaosa, N. Weyl Mott Insulator. *Sci. Rep.* **6**, 19853; doi: 10.1038/srep19853 (2016).

## Supplementary Material

Supplementary Information

## Figures and Tables

**Figure 1:**
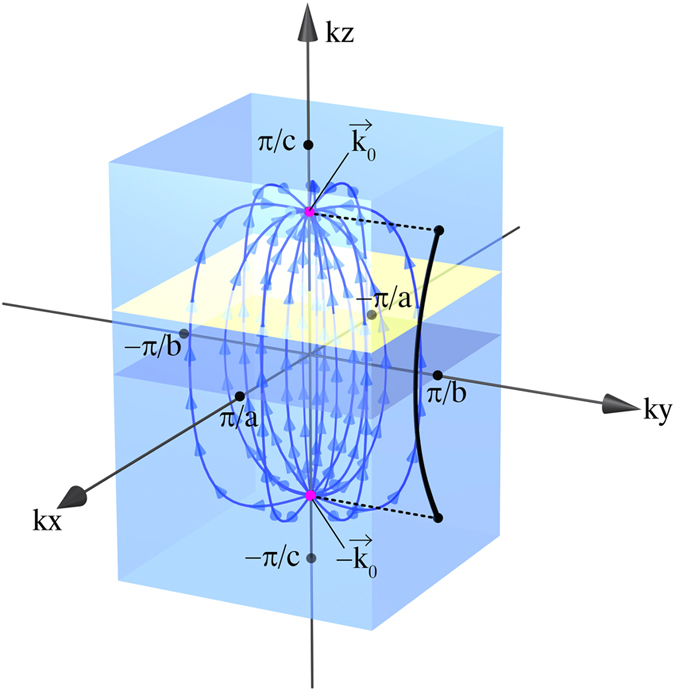
Schematic picture for the Weyl semimetal. A Weyl point plays a role of a source or sink of the Berry curvature, i.e., the (anti-)monopole in the momentum space. A pair of Weyl points with opposite charges is accompanied with a Fermi arc (the curve on the right side surface).
