# Supplementary material for: Weyl Mott Insulator
Source: Sci Rep. 2016 Jan 29;6:19853. doi: 10.1038/srep19853 (PMC4731769; doi:10.1038/srep19853)
Supplement: Supplementary Information [file srep19853-s1.pdf]

Supplementary Information for "Weyl Mott insulator"

Takahiro Morimoto and Naoto Nagaosa

SI. GREEN'S FUNCTION

We derive the Green's function for the Hamiltonian given by

$$H = \psi^\dagger_{\boldsymbol{k}} \boldsymbol{h}(\boldsymbol{k}) \cdot \boldsymbol{\sigma} \psi_{\boldsymbol{k}} + \frac{1}{2} U (n_{\boldsymbol{k}} - 1)(n_{\boldsymbol{k}} - 1), \tag{S1}$$

where

$$\psi_{\boldsymbol{k}} = \begin{pmatrix} c_{\boldsymbol{k}\uparrow} \\ c_{\boldsymbol{k}\downarrow} \end{pmatrix}, \tag{S2}$$

σ are Pauli matrices acting on spin degrees of freedom, $n_{\boldsymbol{k}}$ is the density operator $n_{\boldsymbol{k}} = \psi^\dagger_{\boldsymbol{k}} \psi_{\boldsymbol{k}}$, and U is the magnitude of the repulsive interaction. The repulsive interaction is infinite-ranged in the real space, which can be represented in a local way in the momentum space. Thanks to the locality in the momentum space, the Green's function can be exactly computed for this Hamiltonian as follows.

First we perform a unitary transformation

$$\begin{pmatrix} c_{\boldsymbol{k}\uparrow} \\ c_{\boldsymbol{k}\downarrow} \end{pmatrix} = U(\boldsymbol{k}) \begin{pmatrix} b_{\boldsymbol{k}+} \\ b_{\boldsymbol{k}-} \end{pmatrix} \tag{S3}$$

that diagonalizes the single-particle part of the Hamiltonian as

$$U^\dagger(\boldsymbol{k}) [\boldsymbol{h}(\boldsymbol{k}) \cdot \boldsymbol{\sigma}] U(\boldsymbol{k}) = h(\boldsymbol{k}) \sigma_z. \tag{S4}$$

Then the Green's function is transformed as

$$G_{\alpha\beta}(\boldsymbol{k}, \tau) \equiv -\langle T_\tau c_{\boldsymbol{k}\alpha}(\tau) c^\dagger_{\boldsymbol{k}\beta} \rangle = -U_{\alpha a}(\boldsymbol{k}) U_{\beta a'}(\boldsymbol{k}) \langle T_\tau b_{\boldsymbol{k}a}(\tau) b^\dagger_{\boldsymbol{k}a'} \rangle, \tag{S5}$$

where T_τ denotes the time ordering and $c_{\boldsymbol{k}\alpha}(\tau) = e^{\tau H} c_{\boldsymbol{k}\alpha} e^{-\tau H}$.

In this basis, the Hamiltonian is diagonalized as

$$H|0\rangle = \frac{U}{2}|0\rangle, \tag{S6}$$

$$H b^\dagger_{\boldsymbol{k}-}|0\rangle = -h(\boldsymbol{k}) b^\dagger_{\boldsymbol{k}-}|0\rangle, \tag{S7}$$

$$H b^\dagger_{\boldsymbol{k}+}|0\rangle = h(\boldsymbol{k}) b^\dagger_{\boldsymbol{k}+}|0\rangle, \tag{S8}$$

$$H b^\dagger_{\boldsymbol{k}-} b^\dagger_{\boldsymbol{k}+}|0\rangle = \frac{U}{2} b^\dagger_{\boldsymbol{k}-} b^\dagger_{\boldsymbol{k}+}|0\rangle, \tag{S9}$$

where $|0\rangle$ is the vacuum state and $h(\boldsymbol{k}) = |\boldsymbol{h}(\boldsymbol{k})|$. Thus the expectation value is written as

$$\begin{aligned}
\langle b_{\boldsymbol{k}a}(\tau) b^\dagger_{\boldsymbol{k}a'}\rangle = \frac{1}{Z}[&\langle 0|b_{\boldsymbol{k}a}(\tau) b^\dagger_{\boldsymbol{k}a'}|0\rangle e^{-\beta\frac{U}{2}} \\
&+ \langle 0|b_{\boldsymbol{k}-} b_{\boldsymbol{k}a}(\tau) b^\dagger_{ka'} b^\dagger_{\boldsymbol{k}-}|0\rangle e^{\beta h(\boldsymbol{k})} \\
&+ \langle 0|b_{\boldsymbol{k}+} b_{\boldsymbol{k}a}(\tau) b^\dagger_{\boldsymbol{k}a'} b^\dagger_{\boldsymbol{k}+}|0\rangle e^{-\beta h(\boldsymbol{k})} \\
&+ \langle 0|b_{\boldsymbol{k}+} b_{\boldsymbol{k}-} b_{ka}(\tau) b^\dagger_{\boldsymbol{k}a'} b^\dagger_{\boldsymbol{k}-} b^\dagger_{\boldsymbol{k}+}|0\rangle e^{-\beta\frac{U}{2}}], \qquad \text{(S10)}
\end{aligned}$$

$$Z = e^{\beta h(\boldsymbol{k})} + e^{-\beta h(\boldsymbol{k})} + 2e^{-\beta\frac{U}{2}}. \qquad \text{(S11)}$$

In the right hand side of the equation for $\langle b_{\boldsymbol{k}a}(\tau) b^\dagger_{\boldsymbol{k}a'}\rangle$, the forth term vanishes and other terms are nonzero when $a = a'$. Thus we obtain

$$\begin{aligned}
\langle b_{\boldsymbol{k}+}(\tau) b^\dagger_{\boldsymbol{k}+}\rangle &= \frac{1}{Z}(\langle 0|b_{\boldsymbol{k}+}(\tau) b^\dagger_{\boldsymbol{k}+}|0\rangle e^{-\beta\frac{U}{2}} + \langle 0|b_{\boldsymbol{k}-} b_{\boldsymbol{k}+}(\tau) b^\dagger_{\boldsymbol{k}+} b^\dagger_{\boldsymbol{k}-}|0\rangle e^{\beta h(\boldsymbol{k})}) \\
&= \frac{1}{Z}(e^{\tau(-h+\frac{U}{2})-\beta\frac{U}{2}} + e^{\tau(-h-\frac{U}{2})+\beta h}), \qquad \text{(S12)} \\
\langle b_{\boldsymbol{k}-}(\tau) b^\dagger_{\boldsymbol{k}-}\rangle &= \frac{1}{Z}(\langle 0|b_{\boldsymbol{k}-}(\tau) b^\dagger_{\boldsymbol{k}-}|0\rangle e^{-\beta\frac{U}{2}} + \langle 0|b_{\boldsymbol{k}+} b_{\boldsymbol{k}-}(\tau) b^\dagger_{\boldsymbol{k}-} b^\dagger_{\boldsymbol{k}+}|0\rangle e^{-\beta h(\boldsymbol{k})}) \\
&= \frac{1}{Z}(e^{\tau(h+\frac{U}{2})-\beta\frac{U}{2}} + e^{\tau(h-\frac{U}{2})-\beta h}). \qquad \text{(S13)}
\end{aligned}$$

Therefore, the imaginary-time Green's function is given by

$$\begin{aligned}
G_{++}(\boldsymbol{k}, i\omega_n) &= -\int_0^\beta d\tau e^{i\omega_n\tau}\langle b_{\boldsymbol{k}+}(\tau) b^\dagger_{\boldsymbol{k}+}\rangle \\
&= \frac{1}{Z}\left(\frac{e^{-\beta h} + e^{-\beta\frac{U}{2}}}{i\omega_n - h + \frac{U}{2}} + \frac{e^{\beta h} + e^{-\beta\frac{U}{2}}}{i\omega_n - h - \frac{U}{2}}\right), \qquad \text{(S14)} \\
G_{--}(\boldsymbol{k}, i\omega_n) &= -\int_0^\beta d\tau e^{i\omega_n\tau}\langle b_{\boldsymbol{k}-}(\tau) b^\dagger_{\boldsymbol{k}-}\rangle \\
&= \frac{1}{Z}\left(\frac{e^{-\beta h} + e^{-\beta\frac{U}{2}}}{i\omega_n + h - \frac{U}{2}} + \frac{e^{\beta h} + e^{-\beta\frac{U}{2}}}{i\omega_n + h + \frac{U}{2}}\right). \qquad \text{(S15)}
\end{aligned}$$

A. Green's function for $T = 0$

In the zero temperature ($\beta \to \infty$), the above Green' function reduces to

$$G_{++}(\boldsymbol{k}, i\omega_n) = \frac{1}{i\omega_n - h - \frac{U}{2}}, \qquad \text{(S16)}$$

$$G_{--}(\boldsymbol{k}, i\omega_n) = \frac{1}{i\omega_n + h + \frac{U}{2}}. \qquad \text{(S17)}$$

In the original basis, the Green's function is given by

$$G(\boldsymbol{k}, i\omega_n) = \frac{1}{i\omega_n - \left(h + \frac{U}{2}\right)\boldsymbol{n}\cdot\boldsymbol{\sigma}}, \qquad \boldsymbol{n} = \frac{\boldsymbol{h}}{h}. \qquad \text{(S18)}$$

SII. FERMI ARC

In this section, we study the Fermi arc in the WMIs. Nonvanishing topological indices for the WMIs indicate that the Fermi arc remains in the WMIs, which we verify in the following. Since our model [Eq. (S1)] is diagonalized at each $\boldsymbol{k}$-point and hence we can consider an effective two-dimensional model for each k_z-sector when the system is periodic in the z-direction. Because of the bulk-boundary correspondence, we expect that "edge channels" for each k_z form a Fermi arc. More explicitly, one can obtain the surface bound state from the effective Hamiltonian

$$H_{\text{eff}} = \left[h(\boldsymbol{k}) + \frac{U}{2}\right] \boldsymbol{n}(\boldsymbol{k}) \cdot \boldsymbol{\sigma}, \qquad \boldsymbol{h}(\boldsymbol{k}) = v_F(k_x, k_y, k_z), \tag{S19}$$

by replacing the momenta k_x, k_y with the derivatives $-i\partial_x, -i\partial_y$. Away from the plane $k_z = \pm k_{0z}$, the surface state is almost unchanged from the noninteracting case. The nontrivial issue is how the surface state behaves as k_z approaches $\pm k_{0z}$. Specifically, the problem is whether the penetration depth of the surface states diverges or not with $k_z \to \pm k_{0z}$. Intuitively, the finite gap U indicates that the length scale ξ remains finite, i.e., $\xi \cong \hbar v_F/U$. However, it turns out not when one studies the effective Hamiltonian in Eq. (S19) and the asymptotic behavior of the surface bound state as $|x| \to \infty$ (here we assume $k_y = 0$) by tentatively taking the limit of $|k_x| \ll |k_z|$. In this limit, $H_{\text{eff}} \cong (v_F + \frac{U}{2}|k_z|^{-1})[-i\partial_x\sigma^1 + k_z\sigma^3]$, which indicates that the penetration depth diverges with $\xi = |k_z|^{-1}$. In any case, the length scale is determined by $|k_z|^{-1}$ even when we take into account of the higher orders in ∂_x. Therefore, the surface bound states penetrate into the bulk as k_z approaches to $\pm k_{0z}$.

SIII. OPTICAL CONDUCTIVITY

We study the optical conductivity $\sigma(\omega)$ for a single WF described $\boldsymbol{h}(\boldsymbol{k}) = v_F\boldsymbol{k}$. In the following, we set the Fermi velocity $v_F = 1$, which can be always restored by the dimension analysis.

A. Matrix elements

Here we calculate matrix elements that we will need in evaluation of conductivities, i.e., $\langle \pm|\sigma_i|\pm\rangle$. We first parameterize the direction of the momentum as

$$\boldsymbol{n} = (\sin\theta\cos\phi, \sin\theta\cos\phi, \cos\theta). \tag{S20}$$

Then the wave functions that diagonalize the Hamiltonian are written as

$$|+\rangle = \begin{pmatrix} \cos\frac{\theta}{2} \\ e^{i\phi}\sin\frac{\theta}{2} \end{pmatrix}, \qquad |-\rangle = \begin{pmatrix} -\sin\frac{\theta}{2} \\ e^{i\phi}\cos\frac{\theta}{2} \end{pmatrix}. \tag{S21}$$

The matrix elements are given by

$$\langle +|\sigma_x|+\rangle = \sin\theta\cos\phi, \tag{S22}$$

$$\langle -|\sigma_x|-\rangle = -\sin\theta\cos\phi, \tag{S23}$$

$$\langle +|\sigma_x|-\rangle = \cos\theta\cos\phi + i\sin\phi, \tag{S24}$$

$$\langle +|\sigma_y|+\rangle = \sin\theta\sin\phi, \tag{S25}$$

$$\langle +|\sigma_y|-\rangle = -i\cos\phi + \cos\theta\sin\phi. \tag{S26}$$

In the evaluation of the optical conductivity, we need

$$\int \sin\theta d\theta d\phi \langle \pm|\sigma_x|\pm\rangle\langle \pm|\sigma_x|\pm\rangle = \frac{4\pi}{3}, \tag{S27}$$

$$\int \sin\theta d\theta d\phi \langle +|\sigma_x|-\rangle\langle -|\sigma_x|+\rangle = \frac{8\pi}{3}. \tag{S28}$$

In the evaluation of the Hall conductivity as a function of k_z, we need

$$\int d\phi \langle +|\sigma_x|+\rangle\langle +|\sigma_y|+\rangle = 0, \tag{S29}$$

$$\int d\phi \langle +|\sigma_x|-\rangle\langle -|\sigma_y|+\rangle = 2\pi i\cos\theta = 2\pi i\frac{k_z}{k}. \tag{S30}$$

B. Zero temperature

We first focus on the conductivity $\sigma(\omega)$ for the zero temperature. The Green's function is given by

$$G(i\omega_m) = \frac{1}{(i\omega_m)^2 - (k+\frac{U}{2})^2}\left[i\omega_m + \left(k+\frac{U}{2}\right)\boldsymbol{n}\cdot\boldsymbol{\sigma}\right], \tag{S31}$$

with $\boldsymbol{n} = \boldsymbol{k}/|\boldsymbol{k}|$. The optical conductivity is given by

$$\sigma(\omega) = \mathrm{Re}\left[\frac{Q(\omega+i\epsilon)}{-i\omega}\right], \tag{S32}$$

$$Q(i\Omega) = \lim_{\boldsymbol{q}\to\boldsymbol{0}} \int \frac{d^3\boldsymbol{k}}{(2\pi)^3} \sum_{i\omega_m} \mathrm{tr}[G(\boldsymbol{k}, i\omega_m)\sigma_x G(\boldsymbol{k}+\boldsymbol{q}, i\omega_m + i\Omega)\sigma_x]. \tag{S33}$$

The integrand of $Q(i\Omega)$ reads

$$\begin{aligned}
&\sum_{i\omega_m} \mathrm{tr}[G(\boldsymbol{k}, i\omega_m)\sigma_x G(\boldsymbol{k}, i\omega_m + i\Omega)\sigma_x] \\
&= \sum_{i\omega_m} \frac{1}{(i\omega_m + i\Omega)^2 - (k+\frac{U}{2})^2} \frac{1}{(i\omega_m)^2 - (k+\frac{U}{2})^2} \\
&\quad \mathrm{tr}\left[\left(i\omega_m + i\Omega + \left(k+\frac{U}{2}\right)\boldsymbol{n}\cdot\boldsymbol{\sigma}\right)\sigma_x \left(i\omega_m + \left(k+\frac{U}{2}\right)\boldsymbol{n}\cdot\boldsymbol{\sigma}\right)\sigma_x\right] \\
&= \sum_{i\omega_m} \frac{2}{(i\omega_m + i\Omega)^2 - (k+\frac{U}{2})^2} \frac{1}{(i\omega_m)^2 - (k+\frac{U}{2})^2} \left[(i\omega_m + i\Omega)i\omega_m + \left(1+\frac{U}{2k}\right)^2 (k_x^2 - k_y^2 - k_z^2)\right] \\
&= \sum_{i\omega_m} \frac{2}{(i\omega_m + i\Omega)^2 - (k+\frac{U}{2})^2} \frac{1}{(i\omega_m)^2 - (k+\frac{U}{2})^2} \left[(i\omega_m + i\Omega)i\omega_m - \frac{1}{3}\left(k+\frac{U}{2}\right)^2\right].
\end{aligned} \tag{S34}$$

By using the formula

$$\sum_{i\omega_m} \frac{[(i\omega_m + i\Omega)i\omega_m - abc]}{[(i\omega_m + i\Omega)^2 - a^2][(i\omega_m)^2 - b^2]} = \frac{1}{2}\frac{(a+b)(1-c)}{[i\Omega - (a+b)][i\Omega + (a+b)]} \tag{S35}$$

for $a > 0, b > 0$, we perform the summation over $i\omega_m$ for the above equation and obtain

$$\sum_{i\omega_m} \mathrm{tr}[G(\boldsymbol{k}, i\omega_m)\sigma_x G(\boldsymbol{k}, i\omega_m + i\Omega)\sigma_x] = \frac{8}{3}\frac{\left(k+\frac{U}{2}\right)}{[i\Omega - (2k+U)][i\Omega + (2k+U)]}. \tag{S36}$$

After the analytic continuation $i\Omega \to \omega + i\epsilon$, only the pole at $k = \omega - \frac{U}{2}$ contributes to the imaginary part of the k-integral. Thus, we obtain

$$\begin{aligned}
\mathrm{Im}[Q(\omega)] &= \frac{4}{3\pi^2}\int_0^\infty k^2 dk \frac{\left(k+\frac{U}{2}\right)}{\omega + (2k+U)} \mathrm{Im}\left[\frac{1}{\omega + i\epsilon - (2k+U)}\right] \\
&= -\frac{1}{24\pi}(\omega - U)^2\theta(\omega - U),
\end{aligned} \tag{S37}$$

where we used the formula $\mathrm{Im}\left[\frac{1}{k-a-i\epsilon}\right] = \pi\delta(a)$. Hence, the optical conductivity for the zero temperature is given by

$$\sigma(\omega) = -\frac{\mathrm{Im}[Q(\omega)]}{\omega} = \frac{1}{24\pi\omega}(\omega - U)^2\theta(\omega - U). \tag{S38}$$

By restoring the unit of $e^2/\hbar$ and the Fermi velocity v_F, we end up with

$$\sigma(\omega) = \frac{e^2}{12hv_F\omega}(\omega - U)^2\theta(\omega - U). \tag{S39}$$

1. Poles of $\sigma(\boldsymbol{q},\omega)$

Let us study the locus of the poles of the two-particle correlation function that contribute to the conductivity $\sigma(\boldsymbol{q},\omega)$ for nonzero $\boldsymbol{q}$. From Eq. (S35) and setting $a=|\boldsymbol{k}|+\frac{U}{2}$ and $b=|\boldsymbol{k}+\boldsymbol{q}|+\frac{U}{2}$, the poles of $\sum_{i\omega_m}\mathrm{tr}[G(\boldsymbol{k},i\omega_m)\sigma_x G(\boldsymbol{k}+\boldsymbol{q},i\omega_m+i\Omega)\sigma_x]$ can be read off as $\omega=a+b=|\boldsymbol{k}|+|\boldsymbol{k}+\boldsymbol{q}|+U$. By using the formula $|\boldsymbol{k}|+|\boldsymbol{k}+\boldsymbol{q}|\geq|\boldsymbol{q}|$ and restoring the Fermi velocity v_F, the lower bound of the poles is given by

$$\omega=U+v_F|\boldsymbol{q}|. \tag{S40}$$

C. Finite temperature

In this section, we calculate the optical conductivity $\sigma(\omega)$ in the finite temperature. In doing so, we consider contributions from interband and intraband transitions separately as

$$\sigma(\omega)=\sigma^{\mathrm{inter}}(\omega)+\sigma^{\mathrm{intra}}(\omega). \tag{S41}$$

1. Interband transition

The interband contribution to $Q(i\Omega)$ is given by

$$Q_{\mathrm{inter}}(i\Omega)=\frac{1}{(2\pi)^3}\int k^2dkA_{\mathrm{inter}}(i\Omega), \tag{S42}$$

where

$$\begin{aligned}
&A_{\mathrm{inter}}(i\Omega)\\
&\equiv\int\sin\theta d\theta d\phi\sum_{i\omega_m}[G_{++}(\boldsymbol{k},i\omega_m)\langle+|\sigma_x|-\rangle G_{--}(\boldsymbol{k},i\omega_m+i\Omega)\langle-|\sigma_x|+\rangle\\
&\qquad\qquad+G_{++}(\boldsymbol{k},i\omega_m+i\Omega)\langle+|\sigma_x|-\rangle G_{--}(\boldsymbol{k},i\omega_m)\langle-|\sigma_x|+\rangle]\\
&=\frac{8\pi}{3}Z^{-2}\sum_{s=\pm1}\Big[\left(\frac{e^{\beta k}+e^{-\beta\frac{U}{2}}}{2k+U+is\Omega}+\frac{e^{-\beta k}+e^{-\beta\frac{U}{2}}}{2k+is\Omega}\right)e^{-\beta\frac{U}{2}}+\left(\frac{e^{\beta k}+e^{-\beta\frac{U}{2}}}{2k+is\Omega}+\frac{e^{-\beta k}+e^{-\beta\frac{U}{2}}}{2k-U+is\Omega}\right)e^{-\beta k}\\
&\qquad+\left(\frac{e^{\beta k}+e^{-\beta\frac{U}{2}}}{-(2k+U)+is\Omega}+\frac{e^{-\beta k}+e^{-\beta\frac{U}{2}}}{-2k+is\Omega}\right)e^{\beta k}+\left(\frac{e^{\beta k}+e^{-\beta\frac{U}{2}}}{-2k+is\Omega}+\frac{e^{-\beta k}+e^{-\beta\frac{U}{2}}}{-(2k-U)+is\Omega}\right)e^{-\beta\frac{U}{2}}\Big].
\end{aligned} \tag{S43}$$

This is reduced to

$$Q_{\mathrm{inter}}(\omega) = \frac{1}{3\pi^2} Z^{-2} \sum_{s=\pm 1} \int k^2 dk \left(\frac{e^{\beta k} + e^{-\beta \frac{U}{2}}}{2k + U + is\Omega} + \frac{e^{-\beta k} + e^{-\beta \frac{U}{2}}}{2k + is\Omega} \right) (-e^{\beta k} + e^{-\beta \frac{U}{2}}) + \left(\frac{e^{\beta k} + e^{-\beta \frac{U}{2}}}{2k + is\Omega} + \frac{e^{-\beta k} + e^{-\beta \frac{U}{2}}}{2k - U + is\Omega} \right) (e^{-\beta k} - e^{-\beta \frac{U}{2}}). \tag{S44}$$

After the analytic continuation $i\Omega \to \omega + i\epsilon$, poles that contribute to the imaginary part of the k-integral are

$$k = \frac{\omega - U}{2}, \frac{\omega}{2}, \frac{\omega + U}{2}, \frac{U - \omega}{2}. \tag{S45}$$

Thus the interband contribution to the optical conductivity at the finite temperature is given by

$$\begin{aligned} \sigma^{\mathrm{inter}}(\omega) &= \mathrm{Im}\left[\frac{Q_{\mathrm{inter}}(\omega)}{-i\omega} \right] \\ &= -\frac{1}{6\pi\omega} \Big[(e^{\beta \frac{\omega - U}{2}} + e^{-\beta \frac{U}{2}})(-e^{\beta \frac{\omega - U}{2}} + e^{-\beta \frac{U}{2}}) \left(\frac{\omega - U}{2} \right)^2 Z \left(\frac{\omega - U}{2} \right)^{-2} \theta(\omega - U) \\ &+ 2(e^{-\beta \frac{\omega + U}{2}} - e^{\beta \frac{\omega - U}{2}}) \left(\frac{\omega}{2} \right)^2 Z \left(\frac{\omega}{2} \right)^{-2} \\ &+ (e^{-\beta \frac{\omega + U}{2}} + e^{-\beta \frac{U}{2}})(e^{-\beta \frac{\omega + U}{2}} - e^{-\beta \frac{U}{2}}) \left(\frac{\omega + U}{2} \right)^2 Z \left(\frac{\omega + U}{2} \right)^{-2} \\ &- (e^{-\beta \frac{-\omega + U}{2}} + e^{-\beta \frac{U}{2}})(e^{-\beta \frac{-\omega + U}{2}} - e^{-\beta \frac{U}{2}}) \left(\frac{-\omega + U}{2} \right)^2 Z \left(\frac{-\omega + U}{2} \right)^{-2} \theta(-\omega + U) \Big]. \end{aligned} \tag{S46}$$

The minus sign for the term in the last line arises because the pole $k = \frac{U - \omega - i\epsilon}{2}$ locates in the lower half plane while other poles locate in the upper half plane.

2. *Intraband transition*

The intraband contribution to $Q(i\Omega)$ is given by

$$Q_{\mathrm{intra}}(i\Omega) = \frac{1}{(2\pi)^3} \int k^2 dk [A_{\mathrm{intra}}(i\Omega) + B_{\mathrm{intra}}(i\Omega)], \tag{S47}$$

where

$$\begin{aligned}A_{\rm intra}(i\Omega) &\equiv \int \sin\theta d\theta d\phi \sum_{i\omega_n} G_{++}(k,i\omega_n)\langle+|\sigma_x|+\rangle G_{++}(k+q,i\omega_n+i\Omega_m)\langle+|\sigma_x|+\rangle \\ &= \frac{4\pi}{3}\frac{1}{Z^2}\Big[(e^{-\beta h}+e^{-\beta\frac{U}{2}})^2\frac{n_F(h_k-\frac{U}{2})-n_F(h_{k+q}-\frac{U}{2})}{i\Omega+h_k-h_{k+q}} \\ &+(e^{\beta h}+e^{-\beta\frac{U}{2}})(e^{-\beta h}+e^{-\beta\frac{U}{2}})\frac{n_F(h_k-\frac{U}{2})-n_F(h_{k+q}+\frac{U}{2})}{i\Omega+h_k-h_{k+q}-U} \\ &+(e^{\beta h}+e^{-\beta\frac{U}{2}})^2\frac{n_F(h_k+\frac{U}{2})-n_F(h_{k+q}+\frac{U}{2})}{i\Omega+h_k-h_{k+q}} \\ &+(e^{\beta h}+e^{-\beta\frac{U}{2}})(e^{-\beta h}+e^{-\beta\frac{U}{2}})\frac{n_F(h_k+\frac{U}{2})-n_F(h_{k+q}-\frac{U}{2})}{i\Omega+h_k-h_{k+q}+U}\Big],\end{aligned} \tag{S48}$$

and

$$\begin{aligned}B_{\rm intra}(i\Omega) &\equiv \int \sin\theta d\theta d\phi \sum_{i\omega_n} G_{--}(k,i\omega_n)\langle-|\sigma_x|-\rangle G_{--}(k+q,i\omega_n+i\Omega_m)\langle-|\sigma_x|-\rangle \\ &= \frac{4\pi}{3}\frac{1}{Z^2}\Big[(e^{\beta h}+e^{-\beta\frac{U}{2}})^2\frac{n_F(-h_k-\frac{U}{2})-n_F(-h_{k+q}-\frac{U}{2})}{i\Omega-h_k+h_{k+q}} \\ &+(e^{\beta h}+e^{-\beta\frac{U}{2}})(e^{-\beta h}+e^{-\beta\frac{U}{2}})\frac{n_F(-h_k-\frac{U}{2})-n_F(-h_{k+q}+\frac{U}{2})}{i\Omega-h_k+h_{k+q}-U} \\ &+(e^{-\beta h}+e^{-\beta\frac{U}{2}})^2\frac{n_F(-h_k+\frac{U}{2})-n_F(-h_{k+q}+\frac{U}{2})}{i\Omega-h_k+h_{k+q}} \\ &+(e^{\beta h}+e^{-\beta\frac{U}{2}})(e^{-\beta h}+e^{-\beta\frac{U}{2}})\frac{n_F(-h_k+\frac{U}{2})-n_F(-h_{k+q}-\frac{U}{2})}{i\Omega-h_k+h_{k+q}+U}\Big].\end{aligned} \tag{S49}$$

After performing an analytic continuation $i\Omega_m \to \omega + i\epsilon$ and taking a limit $q \to 0$, we obtain the intraband contribution to the optical conductivity

$$\begin{aligned}&\sigma^{\rm intra}(\omega) \\ &= \frac{1}{6\pi^2}\int dk k^2 \frac{1}{Z(k)^2}\left\{-(e^{-\beta h}+e^{-\beta\frac{U}{2}})^2 n_F'\left(h_k-\frac{U}{2}\right)-(e^{\beta h}+e^{-\beta\frac{U}{2}})^2 n_F'\left(h_k+\frac{U}{2}\right)\right\}\delta(\omega) \\ &\quad+\frac{1}{6\pi^2}\int dk k^2\frac{1}{Z(k)^2}(e^{\beta h}+e^{-\beta\frac{U}{2}})(e^{-\beta h}+e^{-\beta\frac{U}{2}}) \\ &\qquad\times\frac{1}{U}\left[n_F\left(h_k-\frac{U}{2}\right)-n_F\left(h_k+\frac{U}{2}\right)+n_F\left(-h_k-\frac{U}{2}\right)-n_F\left(-h_k+\frac{U}{2}\right)\right]\delta(\omega-U).\end{aligned} \tag{S50}$$

We note that we used the equation $n_F'(\epsilon) = n_F'(-\epsilon)$ in the first term, and the forth term in Eq. (S49) can be discarded after analytic continuation because of a factor $\delta(\omega + U)$.

We show the temperature dependence of weights of peaks at $\omega = 0$ and U in Fig. S1.

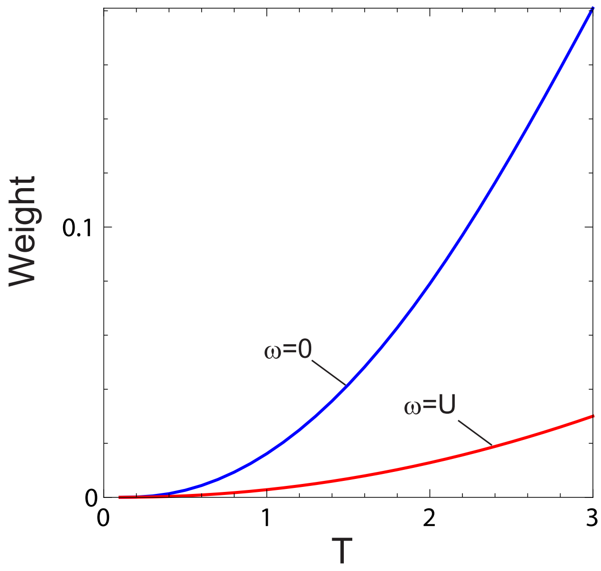

FIG. S1. Temperature dependence of weights of intraband transitions at $\omega = 0$ (blue) and $\omega = U$ (red).

D. Temperature dependence of Drude weight

We study the behavior of the Drude weight in the limit $T \to 0$. The Drude weight is given by the coefficient of $\delta(\omega)$ in Eq. (S50) as

$$W_{\text{Drude}} = \frac{1}{6\pi^2}\int dk k^2 \frac{1}{Z(k)^2}\left\{-(e^{-\beta h} + e^{-\beta\frac{U}{2}})^2 n'_F\left(h_k - \frac{U}{2}\right) - (e^{\beta h} + e^{-\beta\frac{U}{2}})^2 n'_F\left(h_k + \frac{U}{2}\right)\right\}$$
$$= \frac{\beta}{6\pi^2}e^{-\beta\frac{U}{2}}\int dk k^2 \frac{e^{-\beta k} + e^{\beta k}}{(e^{-\beta k} + e^{\beta k} + 2e^{-\beta\frac{U}{2}})^2}. \tag{S51}$$

In the noninteracting case ($U = 0$), the Drude weight behaves as $W_{\text{Drude}} \propto T^2$. This is obtained from a crude estimation by replacing the factor $\frac{e^{-\beta k}+e^{\beta k}}{(e^{-\beta k}+e^{\beta k}+2)^2}$ in the integrand with 1 for $k < T$ and with 0 otherwise. On the other hand, in the case of strong interactions ($U \to \infty$), the Drude weight behaves as $W_{\text{Drude}} \propto e^{-\beta\frac{U}{2}}T^2$. Thus the Drude weight is suppressed exponentially as the interaction U increases.

E. Hall conductivity

We study the Hall conductivity for a fixed value of k_z. The Hall conductivity has a nonzero contribution from a combination

$$\langle T_\tau b_+(\tau)\langle+|\sigma_x|-\rangle b_-^\dagger(\tau)b_-\langle-|\sigma_y|+\rangle b_+^\dagger\rangle. \tag{S52}$$

Here, we set the momentum transfer as $q=0$ because we focus on the dc Hall conductivity. We note that other combinations of current matrices vanish. After we integrate over the direction ϕ of $(k_x,k_y)=k_\parallel(\cos\phi,\sin\phi)$ in current matrices [Eq. (S30)], the expectation value is given by

$$\begin{aligned}Q(\tau) &\equiv \frac{2\pi i k_z}{k}\langle b_+(\tau)b_-^\dagger(\tau)b_-b_+^\dagger\rangle+\langle b_-(\tau)b_+^\dagger(\tau)b_+b_-^\dagger\rangle\\ &=\frac{2\pi i k_z}{k}\frac{1}{Z}(\langle 0|b_-b_+(\tau)b_-^\dagger(\tau)b_-b_+^\dagger b_-^\dagger|0\rangle e^{\beta h}+\langle 0|b_+b_-(\tau)b_+^\dagger(\tau)b_+b_-^\dagger b_+^\dagger|0\rangle e^{-\beta h})\\ &=\frac{2\pi i k_z}{k}\frac{1}{Z}(e^{-2\tau h+\beta h}+e^{2\tau h-\beta h}).\end{aligned} \tag{S53}$$

With the Fourier transformation, we obtain

$$\begin{aligned}Q(i\omega_n) &= \frac{2\pi i k_z}{k}\frac{1}{Z}\int_0^\beta d\tau e^{i\omega_n\tau}\langle b_+(\tau)b_-^\dagger(\tau)b_-b_+^\dagger\rangle+\langle b_-(\tau)b_+^\dagger(\tau)b_+b_-^\dagger\rangle\\ &=\frac{2\pi i k_z}{k}\frac{1}{Z}\left(\frac{-e^{-\beta h}-e^{\beta h}}{i\omega_n-2h}+\frac{-e^{\beta h}-e^{-\beta h}}{i\omega_n+2h}\right)\\ &=\frac{2\pi i k_z}{k}\frac{1}{Z}(e^{\beta h}+e^{-\beta h})\frac{2i\omega_n}{-(i\omega_n)^2+4h^2}\end{aligned} \tag{S54}$$

By performing analytic continuation and taking the zero frequency limit, we obtain the Hall conductivity

$$\begin{aligned}\sigma_{xy}(k_z) &= \frac{1}{(2\pi)^2}\int k_\parallel dk_\parallel \mathrm{Re}(\frac{Q(\omega)}{-i\omega})\\ &=-\frac{1}{2\pi}\int k_\parallel dk_\parallel\frac{k_z}{2k^3}\frac{e^{\beta h}+e^{-\beta h}}{e^{\beta h}+e^{-\beta h}+2e^{-\beta\frac{U}{2}}}\end{aligned} \tag{S55}$$

where $k_\parallel$ is the radial coordinate for (k_x,k_y) and $k=\sqrt{k_\parallel+k_z^2}$. In the zero temperature limit, the Hall conductivity reduces to

$$\sigma_{xy}(k_z)=-\frac{1}{2\pi}\int_0^\infty k_\parallel dk_\parallel\frac{k_z}{2k^3}=-\frac{1}{2\pi}\frac{k_z}{2k}\bigg|_{k_\parallel=0}^{k_\parallel=\infty}=\frac{1}{4\pi}\mathrm{sgn}(k_z). \tag{S56}$$

If we restore the unit of $e^2/\hbar$, the Hall conductivity is given by

$$\sigma_{xy}(k_z)=\frac{e^2}{2h}\mathrm{sgn}(k_z), \tag{S57}$$

which remains quantized into $\pm e^2/2h$ in the WMI.

SIV. STABILITY OF THE MOTT GAP

We study the stability of the Mott gap against the interaction

$$H_C = \sum_{k,k',q} V(\boldsymbol{q}) c^\dagger_{\boldsymbol{k}+\boldsymbol{q},\sigma} c^\dagger_{\boldsymbol{k'}-\boldsymbol{q},\sigma'} c_{\boldsymbol{k'},\sigma'} c_{\boldsymbol{k},\sigma}. \tag{S58}$$

We consider the self-energy arising in the second order of this interaction,

$$\Sigma(\boldsymbol{k}, i\omega) = \int d^3\boldsymbol{q} \sum_{i\Omega} V(\boldsymbol{q})V(-\boldsymbol{q})G(\boldsymbol{k}+\boldsymbol{q}, i\omega + i\Omega)\Pi(\boldsymbol{q}, i\Omega), \tag{S59}$$

with the density-density correlation function

$$\Pi(\boldsymbol{q}, i\Omega) = d^3\boldsymbol{k'} \sum_{i\omega'} \mathrm{Tr}[G(\boldsymbol{k'}, i\omega')G(\boldsymbol{k'}-\boldsymbol{q}, i\omega' - i\Omega)]. \tag{S60}$$

This is explicitly written as

$$\begin{aligned}\Sigma(\boldsymbol{k}, i\omega) = \int d^3\boldsymbol{q} d^3\boldsymbol{k'} \sum_{i\omega', i\Omega} V(\boldsymbol{q})V(-\boldsymbol{q}) &\frac{(i\omega + i\Omega) + (|\boldsymbol{k}+\boldsymbol{q}| + \frac{U}{2})\boldsymbol{n}(\boldsymbol{k}+\boldsymbol{q})\cdot\boldsymbol{\sigma}}{(i\omega+i\Omega)^2 - (|\boldsymbol{k}+\boldsymbol{q}| + \frac{U}{2})^2} \frac{1}{(i\omega')^2 - (|\boldsymbol{k'}| + \frac{U}{2})^2} \\ &\times \frac{1}{(i\omega' - i\Omega)^2 - (|\boldsymbol{k'}-\boldsymbol{q}| + \frac{U}{2})^2} \\ &\times 2\left[i\omega'(i\omega' - i\Omega) + \left(|\boldsymbol{k'}| + \frac{U}{2}\right)\left(|\boldsymbol{k'}-\boldsymbol{q}| + \frac{U}{2}\right)\boldsymbol{n}(\boldsymbol{k'})\cdot\boldsymbol{n}(\boldsymbol{k'}-\boldsymbol{q})\right].\end{aligned} \tag{S61}$$

If the instability for the Mott gap were present, the gap should close at $\boldsymbol{k} = \boldsymbol{0}$ by the consideration from the rotation symmetry. Therefore, we focus on the self-energy for $\boldsymbol{k} = \boldsymbol{0}$. By summing over Matsubara frequencies and setting $\boldsymbol{k} = \boldsymbol{0}$, we obtain

$$\begin{aligned}\Sigma(\boldsymbol{k} = \boldsymbol{0}, i\omega) = \int d^3\boldsymbol{q} d^3\boldsymbol{k'} \frac{|V(\boldsymbol{q})|^2}{2} &\left(1 - \frac{\boldsymbol{k'}\cdot(\boldsymbol{k'}-\boldsymbol{q})}{|\boldsymbol{k'}||\boldsymbol{k'}-\boldsymbol{q}|}\right) \frac{1}{(i\omega - |\boldsymbol{k'}| - |\boldsymbol{k'}-\boldsymbol{q}| - U)^2 - (|\boldsymbol{q}| + \frac{U}{2})^2} \\ &\times \left[i\omega - |\boldsymbol{k'}| - |\boldsymbol{k'}-\boldsymbol{q}| - U + \left(|q| + \frac{U}{2}\right)\boldsymbol{n}_{\boldsymbol{q}}\cdot\boldsymbol{\sigma}\right].\end{aligned} \tag{S62}$$

After performing an integration over $\boldsymbol{k}'$, the terms $|\boldsymbol{k'}-\boldsymbol{q}|$ and $\boldsymbol{k'}\cdot(\boldsymbol{k'}-\boldsymbol{q})$ no longer have a dependence on the angle of $\boldsymbol{q}$, because they only depend on the relative angle between $\boldsymbol{k}'$ and $\boldsymbol{q}$. Then the only term depending on the angle of $\boldsymbol{q}$ after the $\boldsymbol{k}'$ integration is $\boldsymbol{n}_{\boldsymbol{q}}\cdot\boldsymbol{\sigma}$, which vanishes upon the integration over the angle of $\boldsymbol{q}$. Thus the self-energy $\Sigma(\boldsymbol{k} = \boldsymbol{0}, i\omega)$ is diagonal with respect to the spin degrees of freedom. Furthermore, the imaginary part of $\Sigma(\boldsymbol{k}, \omega)$ (after the analytic continuation) appears only at $\omega = |\boldsymbol{k'}| + |\boldsymbol{k'}-\boldsymbol{q}| + |\boldsymbol{q}| + \frac{3U}{2} \geq \frac{3U}{2}$

and $\omega = |\boldsymbol{k'}| + |\boldsymbol{k'} - \boldsymbol{q}| - |\boldsymbol{q}| + \frac{U}{2} \geq \frac{U}{2}$; The imaginary part of $\Sigma(\boldsymbol{k} = \boldsymbol{0}, \omega)$ is zero for $\omega < \frac{U}{2}$. Therefore, the gap of $\frac{U}{2}$ in the Green's function is stable against the inclusion of the interaction H_C.

We note that the the perturbation theory with respect to $V(\boldsymbol{q})$ is valid because of the absence of the infrared divergence. In the case of the contact quartic interaction $V(\boldsymbol{q}) = V$, we notice that the infrared divergence does not appear for $i\omega = 0$ because of the gap of $\frac{U}{2}$ in the energy denominator. In the case of the repulsive Coulomb interaction $V(\boldsymbol{q}) = \frac{4\pi e^2}{q^2}$, the infrared divergence is also absent, because the density-density correlation function behaves $\Pi(\boldsymbol{q}, i\Omega) \propto q^2$ for small q and Ω, and the integral is convergent around $q = 0$.
